# Association between stages of change for smoking cessation and electronic cigarette use among adult smokers: A nationwide cross-sectional study in Korea

**DOI:** 10.1371/journal.pone.0204244

**Published:** 2018-09-20

**Authors:** Boram Kim, Seunghyun Yoo, Sung-il Cho

**Affiliations:** 1 Bureau of Public Health Policy, Office for Healthcare Policy, Ministry of Health and Welfare, Sejong-si, Korea; 2 Department of Public Health Science, Graduate School of Public Health, and Institute of Health and Environment, Seoul National University, Seoul, Korea; University of Montana, UNITED STATES

## Abstract

This study aimed to investigate whether smokers who are ready to quit are more likely to use electronic cigarette (e-cigarette) than those who are not. The association between the ever and current use of e-cigarettes with the stages of change (SOC) model was examined, which reflects the readiness to quit smoking. Current smokers were categorized based on the SOC (precontemplation, contemplation, and preparation). We investigated the associations between quit attempts, intention to quit, and stage of change with e-cigarette use in the general population and different age groups. We used data from the Korea Community Health Survey that was conducted nationwide in 2014, and 45,378 current smokers were included in the study. Adult smokers were more likely to use e-cigarettes simultaneously in the preparation and contemplation stages than in the precontemplation stage (adjusted odds ratio [AOR] of preparation stage: 2.88 and 95% confidence interval [CI]: 2.26–3.66; AOR of contemplation stage: 1.93 and 95% CI: 1.67–2.24). Ever use of e-cigarette was significantly associated with the contemplation stage in smokers younger than 50 years, while current e-cigarette use was greater in the contemplation stage than in the precontemplation stage at all ages. Both ever and current use of e-cigarettes were significantly related with the preparation stage among all age groups except 50–59 years. Because the use of e-cigarette among smokers may continually increase, public health guidance must be provided to different types of dual users. Previous studies on the association between e-cigarette use and the cognitive and behavioral aspects of smokers have conflicting results. This study highlights whether the use of e-cigarettes can be used as an indicator for the readiness to quit smoking. Thus, counselors can encourage smoking cessation and provide tailored interventions.

## Introduction

Despite the effects of cigarette smoking, numerous countries have not successfully implemented tobacco control [1]. Due to cessation difficulty, smokers usually experience recurrent short-term cessation, and numerous failure attempts are also observed before they succeed quitting [2]. Recently, smokers have been interested with the electronic nicotine delivery system, commonly referred to as electronic cigarettes (e-cigarettes), which have been widely used in numerous countries since its invention in 2003 [3]. It is sometimes believed that e-cigarettes are less harmful than conventional cigarettes and may be helpful in quitting or reducing cigarette use since they can help in smoking cessation as shown in advertisements [4, 5]. It has been argued that the use of e-cigarettes may help smokers quit smoking or reduce tobacco consumption by relieving withdrawal symptoms or replacing some rituals associated with smoking habits [6]. Several studies showed that e-cigarette users were more likely to quit smoking [7–9]. However, studies on the increasing proportion of individuals who perceived e-cigarettes to be equally or more harmful compared to regular cigarettes have conflicting results [10]. Recent studies have shown that e-cigarette use was not associated with long-term cessation of tobacco use [11, 12], and data that can support the efficacy of e-cigarette use are limited [13]. In addition, whether the dual use of e-cigarettes along with conventional cigarettes delays quitting, which leads to the re-normalization of tobacco use, has been a cause of concern [14, 15].

Conflicting research results regarding the efficacy of e-cigarette use raise a question on why smokers use e-cigarettes. There is a concern that e-cigarette use will lead non-smokers to smoke or weaken the willingness of smokers to quit. The idea that e-cigarette use causes the reduced willingness of smokers to quit has been a concern. E-cigarette use may help smokers satisfy their curiosity, smoke in public places, and comply with the recommendations of close individuals [16–18]. The experimental use of e-cigarette is particularly common among young smokers, which is probably associated with sensation seeking or the perception that e-cigarettes are less harmful [19–21]. On the other hand, some dual users have reported that they use e-cigarettes to stop smoking or reduce tobacco consumption [3, 22]. In addition, young smokers use e-cigarettes to help them quit smoking [23].

Although dual users agree on the potential benefit of e-cigarettes as a cessation tool, not all dual users use e-cigarettes for smoking cessation [24]. Evidence on whether smokers use e-cigarettes for different purposes based on their readiness to quit and other factors associated with quitting is limited [3, 21, 22, 25–28]. For instance, age should be considered in the derivation of e-cigarette use because awareness, perception of its harmful effects, and motivation for e-cigarette use can vary with age [17, 29]. However, previous studies that focus on the target specific age groups, particularly young smokers, are limited [21, 26, 27, 30].

Despite the research efforts to determine the relationship between e-cigarette use and quitting behaviors, such as attempt or intention to quit, the results were conflicting [7, 27, 28, 31]. To better understand the readiness of smokers to quit, we conducted a study based on the trans-theoretical model (TTM), which has been applied to various studies of health behavior, including smoking cessation [32]. The stages of change (SOC) model is based on the TTM and is useful for clearly conceptualizing the process by which smokers decide and implement smoking cessation [33, 34]. Assuming that an individual moves through the SOC in a progressive manner, each stage is characterized by the presence of various beliefs, knowledge, and attitudes about the target behavior [33]. The SOC would indicate the addiction level of smokers and predict smoking cessation [34], and it serves as a basis for healthcare providers to identify the smoker’s readiness to quit and provide customized interventions [32]. For this reason, SOC has been included in smoking cessation guidelines in various countries. While there are advantages to being intuitive and simple in the SOC based on TTM, the model has been questioned. The conceptual validity and practicality of the SOC have become an issue [35, 36]. Despite this concern, previous studies suggested a reasonable construct validity of the model [37, 38]. SOC can differentiate smokers who are considering cessation through the use of a more proximal plan and a recent quit attempt together, thereby precisely assessing the readiness to quit [34].

In a previous research, it was shown that smokers’ beliefs and their reasons for using e-cigarette use may differ according to age. Hence, we investigated the relationship between readiness to quit and e-cigarette use in different age groups, which have not been carried out in other studies.

## Methods

### Data source

In the present study, public data from 2014 Korea Community Health Survey (KCHS) conducted nationwide by Korea Centers for Disease Control and Prevention were used. Community-based cross-sectional surveys have been conducted annually according to the information in the registration data. To obtain representative samples of adult populations, a complex sampling design was adopted in KCHS, and trained interviewers collected data from the participants based on a structured questionnaire [39]. The 2014 KCHS was the first nationwide survey on e-cigarette use and behavior factors that affect cessation among the adult population in Korea. Of the 200,000 participants, 45,378 were included in the study. The survey methodology has been described elsewhere [40]. The study used public data from a 2014 Korea Community Health Survey (KCHS) which was approved by Institutional Review Board of Korea Centers for Disease Control and Prevention. And this study was approved by the Seoul National University Institutional Review Board (IRB No: E1610/03-04).

### Measures

Current smokers were defined as participants who smoked every day or occasionally and had smoked at least 100 cigarettes in their lifetime. Ever use of an e-cigarette was assessed with the question “Have you ever tried e-cigarettes?” In addition, current use of e-cigarettes was assessed with the question “Do you use e-cigarettes currently?” All responses were obtained as a dichotomous variable (yes or no). Quit attempts were assessed with the question “Have you ever quit smoking more than a day (24 hours)?” Categorical response options for the question included: None, before 1 year ago, and within the past 1 year. Intention to quit was defined as a response to the question “Do you have any plan to quit smoking?”. Categorical response options for the question included: None, someday, within the next 6 months, and within the next 30 days.

Data on the attempts and intention to quit were used to assign smokers to the appropriate stage of change (precontemplation, contemplation, and preparation) [34]. Smokers who did not consider quitting smoking within the next 6 months were classified in the precontemplation stage [34]. Those with the intention to quit within next 6 months but had not attempted in the past year were assigned in the contemplation stage. Those who reported an intention to quit within the next 30 days and attempted to quit within the last year were assigned in the preparation stage. The criteria for defining each stage based on time period in the SOC was controversial [35, 36]. However, evidence supporting the construct validity of the model has shown that SOC is comparable to other relevant instruments [37]. In addition, past quit attempts is identified as one of the important factors in predicting future attempts [41], thus could strengthen the predictive validity of this model.

Participants’ characteristics included socio-demographic factors (sex, age, marital status, education, and income) and smoking- and cessation-related variables (alcohol consumption, self-reported stress, residence in districts with smoke-free regulation, age of smoking commencement, cigarettes smoked per day, and family smoking status).

### Statistical analyses

We conducted an analysis of procedures using weighted factors based on the sample design of the KCHS structure. The participants’ characteristics were expressed as numbers (not weighted) and percentage with standard error (weighted). Multivariable logistic regression was used to investigate the associations between quit attempts, intention to quit, and stage of change with e-cigarette use. Multivariable logistic regression was also performed to determine the associations between SOC and e-cigarette use among different age groups (19–29, 30–39, 40–49, 50–59, and ≥60). The odds ratio (OR) and 95% confidence interval (CI) were calculated for unadjusted and multivariable adjusted models. All analyses were performed using PASW version 18.0, and a *p* value <0.05 was considered statistically significant.

## Results

### Baseline characteristics of the participants

The study included 45,378 current smokers who completed the interview, and their socio-demographic characteristics are shown in Table 1. About 40.3% of smokers attempted to quit before 1 year ago, and 26.8% attempted to quit within the past 1 year. A total of 19.2% smokers indicated that they would quit within the next 6 months, and 8.5% reported that they would quit within the next 30 days. A majority of smokers were included in the precontemplation stage (72.3%), and the remaining smokers were classified as follows: the contemplation stage (22.5%) and the preparation stage (5.2%). The distribution of socio-demographic factors varies depending on the SOC. In particular, the proportion of the younger age group in the precontemplation stage was smaller than that of individuals in the contemplation and/or preparation stage.

**Table 1:** Baseline characteristics of the participants.

Characteristics	Total		Precontemplation		Contemplation		Preparation	
	N^a^	% (SE)^b^	N^a^	% (SE)^b^	N^a^	% (SE)^b^	N^a^	% (SE)^b^
Sex								
Male	41,544	92.6 (0.1)	31,458	92.6(0.2)	8,182	93.3(0.3)	1,877	91.2(0.7)
Female	3,834	7.4 (0.1)	3,042	7.4(0.2)	614	6.7(0.3)	188	8.8(0.7)
Age (years)								
19–29	5,100	17.4 (0.2)	3,335	15.2(0.3)	1432	22.8(0.6)	333	23.1(1.1)
30–39	9,146	23.8 (0.3)	6,313	22.0(0.3)	2297	28.2(0.6)	536	29.0(1.1)
40–49	11,627	26.8 (0.3)	8,849	27.4(0.3)	2317	26.1(0.5)	461	21.6(1.0)
50–59	9,847	20.0 (0.2)	7,925	21.8(0.3)	1571	15.3(0.4)	351	15.9(1.0)
≥60	9,658	12.1 (0.2)	8,095	13.6(0.2)	1179	7.5(0.3)	384	10.5(0.6)
Marital status								
Single	9,733	28.0 (0.3)	7,003	26.5(0.3)	2,234	32.1(0.6)	496	31.4(1.2)
Married	30,467	63.3 (0.3)	23,222	64.0(0.3)	5,851	61.6(0.6)	1,394	61.7(1.3)
Widowed, divorced or other	5,178	8.7 (0.2)	4,292	9.5(0.2)	711	6.3(0.3)	175	6.9(0.6)
Education								
Elementary school or lower	6,178	7.0 (0.1)	5,409	8.3(0.2)	600	3.7(0.2)	169	3.7(0.3)
Middle school	4,826	7.7 (0.1)	4,021	8.7(0.2)	652	5.1(0.3)	153	5.1(0.5)
High school	17,122	37.2 (0.3)	13,348	39.3(0.3)	3,144	33.6(0.6)	630	28.8(1.1)
College/university or higher	17,252	48.1 (0.3)	11,739	44.1(0.3)	4,400	57.6(0.6)	1,113	62.4(1.2)
Income (million KRW)								
<1	7,604	10.3 (0.2)	6,312	11.3(0.2)	1,012	7.4(0.3)	280	8.7(0.6)
1–2	7,939	14.8 (0.2)	6,278	15.3(0.2)	1,353	13.8(0.4)	308	12.7(0.9)
2–3	9,974	22.6 (0.3)	7,478	22.4(0.3)	2,046	23.1(0.5)	450	22.1(1.0)
3–4	8,081	20.0 (0.3)	5,957	19.7(0.3)	1,754	21.3(0.5)	370	18.6(1.0)
≥4	11,780	32.4 (0.3)	8,492	31.4(0.3)	2,631	34.4(0.6)	657	37.9(1.3)
Alcohol consumption (No. of times/week)								
None	6,286	9.7 (0.2)	5,160	10.6(0.2)	901	7.5(0.3)	225	6.9(0.5)
≤1	18,140	44.3 (0.3)	13,039	42.2(0.3)	4,045	48.9(0.6)	1,056	53.9(1.3)
≥2	20,952	46.0 (0.3)	16,318	47.2(0.3)	3,850	43.5(0.6)	784	39.2(1.3)
Self-reported stress^c^								
Low	30,520	64.6 (0.3)	23,389	65.1(0.3)	5,771	63.2(0.6)	1,360	62.6(1.2)
High	14,858	35.4 (0.3)	11,128	34.9(0.3)	3,025	36.8(0.6)	705	37.4(1.2)
Residence in districts with smoke-free regulation^d^								
No	18,319	23.3 (0.2)	14,410	24.3(0.2)	3,177	21.4(0.5)	732	18.6(0.8)
Yes	27,059	76.7 (0.2)	20,107	75.7(0.2)	5,619	78.6(0.5)	1,333	81.4(0.8)
Age of smoking commencement (year)								
<19	14,896	35.2 (0.3)	11,264	35.2(0.3)	2,951	35.4(0.6)	681	35.2(1.2)
≥19	30,482	64.8 (0.3)	23,253	64.8(0.3)	5,845	64.6(0.6)	1,384	64.8(1.2)
Cigarettes smoked per day								
<10	7,890	17.8 (0.2)	5,254	14.9(0.2)	1,757	20.9(0.5)	879	44.2(1.3)
10–20	17,038	40.7 (0.3)	12,558	39.6(0.3)	3,765	45.5(0.6)	715	35.4(1.2)
20–40	19,060	39.1 (0.3)	15,502	42.7(0.3)	3,110	32.4(0.6)	448	19.2(0.9)
≥40	1,390	2.3 (0.1)	1,203	2.8(0.1)	164	1.2(0.1)	23	1.2(0.3)
Family smoking status^e^								
None	41,767	91.1 (0.2)	31,784	91.2(0.2)	8,082	90.6(0.4)	1,901	91.3(0.8)
Any	3,611	8.9 (0.2)	2,733	8.8(0.2)	714	9.4(0.4)	164	8.7(0.8)
Quit attempts			-	-	-	-	-	-
None	15,702	32.8 (0.3)						
Before 1 year ago	18,884	40.3 (0.3)						
Within the past 1 year	10,792	26.8 (0.3)						
Intention to quit			-	-	-	-	-	-
None	13,058	25.5 (0.3)						
Someday	21,459	46.8 (0.3)						
Within the next 6 months	7,382	19.2 (0.2)						
Within the next 30 days	3,479	8.5 (0.2)						
Stage of change			-	-	-	-	-	-
Precontemplation	34,517	72.3 (0.3)						
Contemplation	8,796	22.5 (0.3)						
Preparation	2,065	5.2 (0.1)						

^a^Data were not weighted (total N = 45,378).

^b^Data were weighted to yield nationally representative estimates.

^c^Self-reported stress was categorized as ‘low’ for participants who felt seldom or a little stressed, and ‘high’ in participants who experienced too much stress in daily.

^d^Districts with smoke-free regulation are defined as municipalities that introduced comprehensive smoke-free regulation at least two years before the survey.

^e^Questionnaire on whether someone else in their household routinely smokes in the home indoor.

SE, standard error; KRW, Korean won.

### Associations of the smokers’ quit attempts and intention to quit and stages of change with e-cigarette use

The results of the multivariable logistic regression analyses are shown in Table 2. Smokers who attempted to quit before 1 year ago were more likely to have tried e-cigarettes or use them currently than those who had never attempted to quit. For those who attempted to quit smoking within a year, significantly larger odds were obtained in both ever and current use. Participants who intended to quit someday were more likely to have tried e-cigarettes or use them currently than those who did not plan to quit. Smokers who intended to quit within the next 6 months or 30 days were significantly more likely to have tried e-cigarettes or use them currently compared to those who intended to quit someday. After adjusting for all other factors, smokers in the contemplation stage were more likely to use e-cigarettes compared to those in the precontemplation stage (adjusted OR [AOR] of ever use: 1.54, 95% CI: 1.44–1.64; AOR of current use: 1.93, 95% CI: 1.67–2.24; p<0.001). Smokers in the preparation stage were more likely to have tried e-cigarettes compared to those in the precontemplation stage (AOR: 1.63; 95% CI: 1.43–1.86). However, no significant difference was observed between the contemplation stage and preparation stage in ever use of e-cigarettes. On the other hand, smokers in the preparation stage were more likely to be current users of e-cigarettes compared with those in both the precontemplation and contemplation stages (AOR: 2.88; 95% CI: 2.26–3.66).

**Table 2:** Associations of the smokers’ quit attempts and intention to quit and stages of change with e-cigarette use.

	Ever use		Current(dual) use	
	Unadjusted OR (95% CI)	Adjusted OR (95% CI)^a^	Unadjusted OR (95% CI)	Adjusted OR (95% CI)^a^
Quit attempts				
None	1.00 (Ref)	1.00 (Ref)	1.00 (Ref)	1.00 (Ref)
Before 1 year ago	1.75 (1.63–1.88)^b^	1.95 (1.81–2.10)^b^	1.40 (1.17–1.68)^b^	1.48 (1.23–1.79)^b^
Within the past 1 year	2.46 (2.28–2.66)^b^	2.62 (2.42–2.84)^b^	3.29 (2.75–3.93)^b^	2.93 (2.43–3.52)^b^
Intention to quit				
None	1.00 (Ref)	1.00 (Ref)	1.00 (Ref)	1.00 (Ref)
Someday	1.56 (1.45–1.68)^b^	1.55 (1.44–1.68)^b^	1.60 (1.30–1.97)^b^	1.49 (1.21–1.83)^b^
Within the next 6 months	2.37 (2.18–2.58)^b^	2.13 (1.95–2.32)^b^	3.28 (2.65–4.06)^b^	2.54 (2.04–3.15)^b^
Within the next 30 days	1.97 (1.76–2.20)^b^	2.06 (1.83–2.32)^b^	3.93 (3.06–5.05)^b^	3.42 (2.64–4.43)^b^
Stage of change				
Precontemplation	1.00 (Ref)	1.00 (Ref)	1.00 (Ref)	1.00 (Ref)
Contemplation	1.67 (1.57–1.79)^b^	1.54 (1.44–1.64)^b^	2.32 (2.01–2.68)^b^	1.93 (1.67–2.24)^b^
Preparation	1.58 (1.39–1.79)^b^	1.63 (1.43–1.86)^b^	3.35 (2.65–4.22)^b^	2.88 (2.26–3.66)^b^

^a^Multivariable logistic regression model adjusted for sex, age group, marital status, education, income, alcohol consumption, self-reported stress, residence in districts with smoke-free regulation, age of smoking commencement, cigarettes smoked per day, family smoking status.

^b^*p*-value<0.001.

OR, odds ratio, CI, Confidence Interval.

### Associations between the stages of change and e-cigarette use by age groups

After adjusting for all other variables, smokers younger than 50 years in the contemplation stage were more likely to use e-cigarettes at least once in their lifetime compared to those in the precontemplation stage (Table 3). Smokers in the contemplation stage were more likely to be current users of e-cigarettes compared to those in the precontemplation stage among all age groups. The adjusted OR of ever and current use among smokers in the preparation stage was higher than that in the precontemplation stage, except for the group that included individuals aged 50–59 years. In addition, smokers who were aged 19–29 years and included in the preparation stage were more likely to use e-cigarettes currently than those in both the precontemplation and contemplation stages. However, no significant differences were observed between the different groups in the preparation and contemplation stages.

**Table 3:** Associations between the stages of change and e-cigarette use by age groups.

	Ever use		Current(dual) use	
	Unadjusted OR (95% CI)	Adjusted OR (95% CI)^a^	Unadjusted OR (95% CI)	Adjusted OR (95% CI)^a^
19–29				
Precontemplation	1.00 (Ref)	1.00 (Ref)	1.00 (Ref)	1.00 (Ref)
Contemplation	1.58 (1.43–1.75)^b^	1.75 (1.57–1.95)^b^	2.05 (1.68–2.50)^b^	2.11 (1.73–2.58)^b^
Preparation	1.52 (1.22–1.89)^b^	1.94 (1.54–2.44)^b^	3.47 (2.45–4.90)^b^	3.66 (2.61–5.14)^b^
30–39				
Precontemplation	1.00 (Ref)	1.00 (Ref)	1.00 (Ref)	1.00 (Ref)
Contemplation	1.51 (1.36–1.66)^b^	1.53 (1.38–1.70)^b^	1.77 (1.44–2.17)^b^	1.71 (1.39–2.10)^b^
Preparation	1.44 (1.19–1.75)^b^	1.61 (1.31–1.97)^b^	2.68 (1.89–3.81)^b^	2.48 (1.73–3.57)^b^
40–49				
Precontemplation	1.00 (Ref)	1.00 (Ref)	1.00 (Ref)	1.00 (Ref)
Contemplation	1.55 (1.38–1.74)^b^	1.61 (1.43–1.81)^b^	1.98 (1.49–2.64)^b^	1.93 (1.43–2.60)^b^
Preparation	1.24 (0.94–1.64)	1.44 (1.08–1.92)^d^	2.21 (1.26–3.87)^c^	2.18 (1.21–3.93)^c^
50–59				
Precontemplation	1.00 (Ref)	1.00 (Ref)	1.00 (Ref)	1.00 (Ref)
Contemplation	1.05 (0.90–1.23)	1.10 (0.94–1.30)	1.82 (1.24–2.67)^c^	1.94 (1.32–2.86)^c^
Preparation	0.96 (0.69–1.32)	1.13 (0.82–1.56)	0.51 (0.15–1.74)	0.59 (0.17–1.99)
≥60				
Precontemplation	1.00 (Ref)	1.00 (Ref)	1.00 (Ref)	1.00 (Ref)
Contemplation	1.17 (0.98–1.39)	1.18 (0.98–1.43)	2.49 (1.83–3.37)^b^	2.41 (1.77–3.28)^b^
Preparation	1.84 (1.41–2.40)^b^	2.16 (1.63–2.87)^b^	4.05 (2.46–6.66)^b^	4.74 (2.77–8.10)^b^

^a^Multivariable logistic regression model adjusted for sex, marital status, education, income, alcohol consumption, self-reported stress, residence in districts with smoke-free regulation, age of smoking commencement, cigarettes smoked per day, family smoking status.

^b^*p*-value<0.001.

^c^*p*-value<0.01.

^d^*p*-value<0.05.

OR, odds ratio, CI, Confidence Interval.

## Discussion

The current study highlights the associations between e-cigarette use and the readiness to quit using the SOC model. Smokers in the contemplation stage were more likely to use e-cigarette compared to those in the precontemplation stage. Furthermore, smokers in the preparation stage were more likely to use e-cigarettes compared to those in other stages. These results are consistent with previous studies with specific patient populations [42]. The contemplation stage evaluates the benefits and barriers to behavior changes, and the preparation stage corresponds to the process of experimenting with small changes prior to determining the behavior change [32]. Some studies have suggested that e-cigarettes can reduce the difficulty of long-term smoking cessation by alleviating the withdrawal symptoms from nicotine use and providing behavioral cues for smoking habit [17]. Smokers including unsuccessful quitters are open to trying e-cigarettes in the future [43], even if its efficacy and effects are not yet validated. The results of the present study suggested that smokers’ SOC have a significant association with e-cigarette use.

Based on the analysis of the relationship between e-cigarette use and SOC, a difference was observed between those who experienced e-cigarette use in the past and those who are currently using e-cigarette along with the regular cigarette [43]. Ever use of e-cigarettes may indicate various reasons other than quitting purposes, whereas current use is associated with a more explicit purpose or reason [22, 27].

We found that young smokers who were ready to quit smoking were more likely to use e-cigarettes at the same time, although some studies have reported that experimental use is common among young individuals [19–21]. This result is consistent with that of previous studies that involve young smokers [23, 27]. The result may be associated with high awareness of young adults about e-cigarette use than the older individuals [3]. Furthermore, young individuals tend to believe e-cigarettes are less harmful, and they are more willing to use e-cigarettes to support their quit attempts than older individuals [4, 19]. In smokers aged 50–59 years, a significant association was not observed between ever use of e-cigarette and SOC. This was consistent with the result of an earlier study that reported that increasing age indicates a greater likelihood of having used nicotine replacement therapy but not e-cigarettes for smoking cessation [44]. This age group is unlikely to try other tobacco products with conventional cigarettes because the marketing of e-cigarettes primarily targeted young smokers [43]. Both ever and current e-cigarette use were significantly associated with the preparation stage in smokers older than 60 years. This may be because hard-core smokers of old age who failed at cessation with approved methods may have opted for e-cigarettes as an alternative. In a previous research, smoking cessation or health was more likely to be cited as a reason for e-cigarette use in older individuals than in younger ones [20]. A qualitative study found that older smokers were not knowledgeable regarding the effectiveness and safety of e-cigarettes and that they recognized it as a cessation tool [45]. The results of the present study help us better understand the similarities and differences in the motivations of young adults and older adults in using e-cigarettes, suggesting that a tailored intervention to meet their needs [17].

The results of this study are consistent with that those of previous studies, showing that e-cigarette use is significantly related with quit attempts and intention to quit [22, 25, 26, 28]. Our study further confirmed that smokers who made attempts to quit before 1 year ago or had the intention to quit someday were also more likely to use e-cigarettes than the reference group. To the best of our knowledge, previous studies have not obtained similar results [22, 25, 26].

To the best of our knowledge, this study first examined the relationship between SOC and e-cigarette use in the general population. In addition, we conducted an analysis of the subgroups in terms of age, which was not adequately investigated in previous studies. The survey participants were recruited via systemic sampling and data collected through one-on-one interview. Therefore, results of this study can be generalized compared to those of other studies, which were conducted using a small sample size, or obtained from participants using self-reported questionnaires.

However, this study had several limitations. First, a cross-sectional design was used. Therefore, results cannot be interpreted as causal inferences. Information on when participants had used e-cigarettes in the past was not included. Therefore, the ever e-cigarette use could have occurred prior to the respondents’ interest in quitting smoking. Second, this study did not include the analysis of the details of e-cigarette use, such as frequency of use, total duration of use, nicotine dosage and device type. Some studies reported that the frequency and duration of e-cigarette use may be associated with quit attempts or cessation [25], and device characteristics may be related with the behavior of individuals toward e-cigarette use [18]. National health surveys in Korea have not covered the device type of e-cigarettes, it is very necessary that this should be included in future Korean surveys. Lastly, study results cannot be generalized to countries where smoking rates are more balanced across sexes. Because this study included fewer female participants due to the gender difference in smoking prevalence. However, men still account for a most of the total smokers in the world as well as in the major countries with the largest number of smokers [46]. Previous studies have reported no gender difference in the use of e-cigarettes among smokers [30], further studies is needed on various populations with different gender gap in terms of smoking prevalence.

## Conclusion

In conclusion, adult smokers who were ready to quit were more likely to have tried e-cigarettes or use them currently. However, result varied, depending on their age and current status of e-cigarette users. With the current trends, the use of e-cigarettes will continue to increase globally. The present study may provide an implication for future research since it provides information on the proper interventions for dual users who need support.
